# Pangenome graphs in infectious disease: a comprehensive genetic variation analysis of *Neisseria meningitidis* leveraging Oxford Nanopore long reads

**DOI:** 10.3389/fgene.2023.1225248

**Published:** 2023-08-10

**Authors:** Zuyu Yang, Andrea Guarracino, Patrick J. Biggs, Michael A. Black, Nuzla Ismail, Jana Renee Wold, Tony R. Merriman, Pjotr Prins, Erik Garrison, Joep de Ligt

**Affiliations:** ^1^ Institute of Environmental Science and Research, Porirua, New Zealand; ^2^ Department of Genetics, Genomics and Informatics, University of Tennessee Health Science Center, Memphis, TN, United States; ^3^ Genomics Research Centre, Human Technopole, Milan, Italy; ^4^ Molecular Biosciences Group, School of Natural Sciences, Massey University, Palmerston North, New Zealand; ^5^ Molecular Epidemiology and Public Health Laboratory, School of Veterinary Science, Massey University, Palmerston North, New Zealand; ^6^ Department of Biochemistry, University of Otago, Dunedin, New Zealand; ^7^ School of Biological Sciences, University of Canterbury, Christchurch, New Zealand; ^8^ Division of Clinical Immunology and Rheumatology, University of Alabama at Birmingham, Birmingham, AL, United States

**Keywords:** pangenome graphs, infectious diseases, genomic surveillance, comparative genomics, genetic variation, long-read sequencing, genome assembly

## Abstract

Whole genome sequencing has revolutionized infectious disease surveillance for tracking and monitoring the spread and evolution of pathogens. However, using a linear reference genome for genomic analyses may introduce biases, especially when studies are conducted on highly variable bacterial genomes of the same species. Pangenome graphs provide an efficient model for representing and analyzing multiple genomes and their variants as a graph structure that includes all types of variations. In this study, we present a practical bioinformatics pipeline that employs the PanGenome Graph Builder and the Variation Graph toolkit to build pangenomes from assembled genomes, align whole genome sequencing data and call variants against a graph reference. The pangenome graph enables the identification of structural variants, rearrangements, and small variants (e.g., single nucleotide polymorphisms and insertions/deletions) simultaneously. We demonstrate that using a pangenome graph, instead of a single linear reference genome, improves mapping rates and variant calling for both simulated and real datasets of the pathogen *Neisseria meningitidis*. Overall, pangenome graphs offer a promising approach for comparative genomics and comprehensive genetic variation analysis in infectious disease. Moreover, this innovative pipeline, leveraging pangenome graphs, can bridge variant analysis, genome assembly, population genetics, and evolutionary biology, expanding the reach of genomic understanding and applications.

## Introduction

Over the last two decades, whole genome sequencing (WGS) has become an indispensable tool in infectious disease research, surveillance, and control (Didelot et al., 2012; Gardy and Loman, 2018). Rapid advancements in sequencing technologies and bioinformatic analysis have facilitated the generation of high-quality genomic data at an unprecedented scale (Goodwin et al., 2016; Nurk et al., 2022). WGS has enabled researchers to track and monitor the spread and evolution of pathogens, investigate outbreaks, identify drug resistance markers, and develop diagnostic assays and vaccines (Koser et al., 2014; Walker et al., 2015; Quick et al., 2016; Chen et al., 2021; Yang et al., 2021). Its utility has been especially evident in the SARs-CoV-2 pandemic, enabling real-time tracking of the pandemic (Geoghegan et al., 2021) and identification of transmission chains (Geoghegan et al., 2020). Additionally, WGS has provided valuable insights into the genetic diversity, population structure, and functional characteristics of various pathogens, thereby shaping our understanding of the molecular mechanisms driving their virulence and transmission (Holt et al., 2015).

Currently, genomic surveillance concentrates on monitoring lineages and establishing transmission links between cases. Analysis is mainly dependent on mutations in the core genome (the genomic regions that are common to all isolates being analyzed at that time), using one linear genome as a reference. Bacteria genomes are highly variable, with genomic rearrangements and different-scale deletion or insertion events being common (Darmon and Leach, 2014). Using a single reference approach, variations in the accessory genome (regions not shared by all the genomes) are not detected, suggesting we may miss important variations and introduce biases due to the selection of the reference genome. Consequently, the alignment of sequencing data against a single reference genome may lead to inaccurate or incomplete variant identification (Garrison and Math, 2012). Moreover, the linear representation of a genome fails to capture the complexity of genomic rearrangements, duplications, and structural variants (SVs) that are critical for understanding pathogen evolution and adaptation, especially in highly recombinogenic species (Eizenga et al., 2020; Colquhoun et al., 2021). Viruses, responsible for many infectious diseases, possess highly variable genomes that complicate genomic surveillance (Sanjuán and Domingo-Calap, 2016). These tiny pathogens can rapidly evolve and adapt to changing environments, with the potential to jump species barriers, as seen with the emergence of SARS, MERS, and COVID-19 (Xu et al., 2004; Mohd et al., 2016; Plowright et al., 2017; Lu et al., 2020). Viral genomes, particularly those of RNA viruses, are characterized by high mutation rates which can lead to the emergence of new viral strains with altered virulence or transmissibility (Domingo et al., 2021). Accounting for the variability and unique characteristics of viral genomes is essential for comprehensive disease monitoring and management.

To overcome these limitations, pangenome graphs have emerged as an alternative approach for representing and analyzing multiple genomes and their variants (Garrison et al., 2018; Rakocevic et al., 2019; Liao et al., 2023; The Computational Pan-Genomics Consortium, 2018). A pangenome graph is a graph-based data structure that captures the entire genomic diversity of a set of related genomes by incorporating all types of variation, including SVs, rearrangements, and small variants (e.g., single nucleotide polymorphisms (SNPs) and insertions/deletions) (Paten et al., 2017; Marschall et al., 2018). By representing collections of genomes and their alignments as graphs, pangenome graphs allow for more accurate and comprehensive genetic variation analysis, as they provide a unified framework to compare and analyze diverse genomes, overcoming the biases associated with single linear reference genomes (Paten et al., 2017).

Different methods are available for constructing pangenomes, each tailored to suit specific research objectives and employing unique techniques. Minigraph generalizes minimap2, which only calls SVs (Li et al., 2020). Cactus uses a phylogenetic tree to guide the creation of multiple alignments (Armstrong et al., 2020), and the Cactus Pangenome Pipeline adapts Cactus to eliminate the need for a guide tree and adds base-level alignments to the minigraph graph, though it is still single reference-based (Hickey et al., 2023). PPanGGOLiN uses gene families as nodes and genomic neighborhoods as edges (Gautreau et al., 2020), and Pandora focuses on SNPs of pangenomes by constructing graphs based on individual multiple sequence alignments of coding sequences and intergenic regions (Colquhoun et al., 2021). Meanwhile, minimizer-space de Bruijn graphs offer a graph representation for highly accurate, long sequencing reads (Ekim et al., 2021). In contrast to these tools, the PanGenome Graph Builder (PGGB) stands out as the least unbiased method (Garrison et al., 2023). PGGB incorporates an “all-versus-all” alignment method, treating each input genome with equal importance. The graphs produced by PGGB provide a base-level representation of the pangenome, even within repetitive regions, and include variants of all scales, from SNPs to large SVs. This allows every included genome to serve as a reference for subsequent analysis (Garrison et al., 2023). PGGB has previously been used to build the draft human pangenome (Liao et al., 2023), and Guarracino and colleagues have used it to validate a longstanding hypothesis regarding the evolution of human acrocentric chromosomes (Guarracino et al., 2023). Therefore, we used PGGB for pangenome graph construction because of its comprehensive and unbiased capabilities.

The implementation of pangenome graphs in infectious disease research is crucial, offering significant advantages. The use of pangenome graphs not only allows for the identification of novel genetic variants and SVs that may be overlooked by traditional linear reference-based methods (Garrison et al., 2018), but also provides the potential to address some longstanding unresolved questions, such as the origin of antibiotic resistance (Forsberg et al., 2014), the evolution of pathogenicity (Zhou et al., 2020), and the impact of horizontal gene transfer and evolution of genome architecture (Soucy et al., 2015). *Neisseria (N.) meningitidis*, also known as the meningococcus pathogen, is the primary agent responsible for invasive meningococcal diseases such as meningitis and septicemia, causing isolated incidents, outbreaks, and epidemics worldwide (Halperin et al., 2012). The genome of this bacterium spans approximately 2.1–2.4 Mb and possesses a GC content ranging from 51%–52%. One striking characteristic of *Neisseria meningitidis* genomes is their high recombination rate, which largely fuels the extensive genetic diversity within this species (Schoen et al., 2009; Didelot and Maiden, 2010; Harrison et al., 2017). In this study, we utilized both real and simulated genomic data of *N. meningitidis* to assess the pangenome pipeline, covering pangenome graph construction to variant calling. Our findings demonstrated that using pangenome graphs improves mapping rates and enhances variant calling. This heightened accuracy, encompassing all types of variants, has the potential to improve outbreak investigations, predict drug resistance, and facilitate vaccine design (Rasko et al., 2008; Naz et al., 2019). By employing the least unbiased pangenome graph construction tool PGGB and utilizing a graph reference for subsequent NGS data analysis, our pangenome graph pipeline offers a promising and practical approach for comparative genomics and comprehensive genetic variation analysis in infectious disease research. This paves the way for more accurate and in-depth investigations of pathogen diversity, evolution, and adaptation (Paten et al., 2017; Rakocevic et al., 2019).

## Materials and methods

### Background of *Neisseria meningitidis* NZMenB epidemic strain

In Aotearoa New Zealand (NZ), from 1991 to 2007, an extended serogroup B epidemic occurred due to a single strain known as NZMenB (designated B:4:P1.7-2,4), identified by the PorA variant (P1.7-2), which still accounts for around one-third of meningococcal disease cases in NZ (Dyet and Martin, 2006; Yang et al., 2021). Based on our unpublished WGS data, we have categorized NZMenB into three phylogenetic clades, namely, clade154, clade41 and clade42 based on the multilocus sequence types (MLST) of seven housekeeping genes for sequence type (ST), ST154, ST41 and ST42 respectively (Maiden et al., 1998). The epidemic was primarily driven by two monophyletic clades, namely, ST154 and ST42, which accounted for the majority of the disease cases. On the other hand, although fewer isolates were associated with ST41, it displayed greater diversity, with the presence of multiple distinct lineages.

### Nanopore long-reads

To analyze the WGS dataset, the original reference genome NC_017518 (a ST42 isolate) was used. To obtain complete reference genomes for NMI01191 (a ST41 isolate) and NMI97348 (a ST154 isolate), we conducted Nanopore long-read sequencing. High molecular weight genomic DNA was extracted using the Gentra Puregene Yeast/Bact. Kit (QIAGEN) and purified with Agilent Magnetic Beads. We used 400 ng of high molecular weight genomic DNA to construct sequence libraries utilizing the SQK-RBK004 Rapid Barcoding kit (Oxford Nanopore Technologies). The libraries were sequenced on R9.4.1 MinION flow cells. We used Flye version 2.8.1 (Kolmogorov et al., 2019) for *de novo* assembly, and Illumina sequencing reads were employed to polish the assembly using Unicycler version 0.4.8 (Wick et al., 2017). Consequently, we were able to obtain complete NZMenB genomes (3STs) comprising NMI01191 for ST41, NMI97348 for ST154, and NC_017518 for ST42. The 3ST genomes were aligned using progressiveMauve (Darling et al., 2004).

### Simulation of genomes for pangenome graph construction

Mauve alignments demonstrated large inversions among the 3ST genomes. To evaluate pangenome graph construction, we simulated three genomes from NC_017518 (ST42) by introducing either randomly generated SNPs or mutated according to the SNP differences of ST41 and ST154 relative to ST42. The simulation was followed by introducing 200 indels and two inversions using simuG (Yue and Liti, 2019). We named the three simulated genomes ST42Sim, ST41Sim, and ST154Sim. The three simulated genomes contained 200 indels and two inversions relative to ST42, with ST42Sim, ST41Sim and ST154Sim containing 5000, 2892 and 4283 SNPs respectively. We grouped the three simulated genomes with ST42, which we refer to as the 4Sim genomes, and used them for further analysis.

### Downloading diverse *Neisseria meningitidis* genomes from NCBI

To expand our evaluation of pangenome graph construction to more diverse genomes, 130 *N. meningitidis* (NM) genomes were downloaded from NCBI (Supplementary Table S1). The 130NM genomes comprised 8, 20, 20, 62, 2, 13, and 5 of group A, B, C, W, X, Y, and ungrouped, respectively.

### Pangenome graph construction with PGGB

We constructed pangenome graphs for the 4Sim genomes, 3STs of NZMenB, and 130NM genomes using the PanGenome Graph Builder (PGGB) (Garrison et al., 2023). PGGB is a reference-free method for graph construction by employing all-to-all alignments with wfmash, graph induction via seqwish, and progressive normalization using smoothxg and gfaffix, graph visualization and generating statistics using Optimized Dynamic Genome/Graph Implementation (ODGI) (Guarracino et al., 2022; Garrison et al., 2023). To construct the pangenome graphs, we initially aligned the start of ST41 and ST154 with ST42 for the 3STs, and all 130NM genomes were fixed to start with the *dna*A gene using circlator version 1.5.5 (Hunt et al., 2015).

There are three essential parameters for PGGB pangenome graph construction, -n, the number of genomes, -s, the segment length (defines the seed length for alignment used in wfmash), and -p, the minimum pairwise identity between seeds. Here, we explain how we optimized these parameters for our specific datasets. We adopted the mash triangle approach (Ondov et al., 2016) to estimate pairwise distances within each dataset. The maximum distance observed was 0.0038 for the 4Sim genomes, 0.0016 for the 3ST, and 0.0232 for the 130NM. Following the guidance provided by the PGGB developers, we slightly decreased the -p value in accordance with these pairwise distances for more inclusive all-to-all alignments with wfmash. When adjusting -s (1000, 2000, 5000, and 10000) and -p (96, 95 and 90) parameters for the 4Sim genomes, the resulting pangenome graphs were similar across the different parameter settings. Another parameter, -k, influences the graph structure significantly; it excludes matches shorter than a certain threshold from the initial graph model, which we used the default -k 19. The PGGB developers suggest setting -k larger for larger genomes. Larger values for -k also allow us to ignore, when necessary, short homologies due to the intervention of transposable elements, which would increase the complexity of the graph. A lower -p value will result in more inclusive alignments, and a larger -s value can reduce graph complexity by focusing on longer homologies between the genomes being aligned. To finely adjust the PGGB tool for different datasets, these parameters (-s and -p) may require modification based on the specific properties of the genomes, such as their divergence and frequency of SVs. According to the divergence among genomes and known rearrangement in the dataset of 4Sim, 3ST, we set the parameters -s, -p, and -n to 1000, 96, and 4, respectively, for the 4Sim genomes, and to 2000, 95, and 3, respectively, for the 3ST genomes. For the 130NM genomes, we opted for a larger -s 10,000 value, both for scalability reasons and to keep graph complexity lower. As a result, we set the parameters -s, -p, and -n to 10000, 95, and 131, respectively. By employing these selected parameter values, we successfully generated the most concise pangenome graph for each dataset, guaranteeing the optimal alignment of a significant proportion of segments from each path within the graph (https://github.com/pangenome/pgge). Additionally, the “odgi stats -S” option was used to generate statistics for the seqwish and smoothxg graph and “multiqc -m” option was used to generate a MultiQC report of the graphs’ statistics and visualizations. All runs were executed with 48 threads on a Dell R840 server with an Xeon Gold 6244 3.60 GHz CPU with 64 cores, and 3TB RAM at ESR. We also utilized gfaestus for the 2D visualization of the pangenome graphs of both the 4Sim and 130NM (https://github.com/chfi/gfaestus).

### Vg deconstruct to call variants in the graphs

Variation graphs offer a compact representation of genetic variation across a population in the form of bidirected DNA sequence graphs, encompassing large-scale SVs like inversions and duplications (Paten et al., 2017). To identify both small and large variants from the pangenome graph, we employed the Variation Graph (VG) toolkit (Garrison et al., 2018) to deconstruct the variants into VCF files using the path NC_017518 (ST42). The VG toolkit, standing for Variation Graph, enables genomic analysis, such as alignment, assembly, and variant calling, directly on the graph structure, thereby facilitating the study of complex and highly variable genomic regions while maintaining the context of each variation’s position in the genome. When employing the “vg deconstruct” feature, we set the parameters -a to process all snarls (genomic regions containing variant sites and corresponding alternative alleles), including nested ones, -e to consider traversals that correspond to paths in the graph, and -K to retain conflicted genotypes, thereby ensuring the inclusion of all variants present in the graph.

Given that the simulated genomes (ST42Sim, ST41Sim, and ST154Sim) were derived from ST42, the known variations for these simulated genomes relative to ST42 were served as the ground truth. By utilizing this ground truth information, we conducted a comparative analysis, evaluating the observed variations within the 4Sim genome graph. Initially, we filtered for variations larger than 100 bp, and then we utilized vcfallelicprimitives from vcflib v.1.0.0 (Garrison et al., 2022) to deconvolute complex variations that were less than 100 bp. We compared the variants identified in the graph with the established ground truth to evaluate their consistency. Variants were categorized as consistent if they were present in both the graph and the ground truth, as false negatives if they were present in the ground truth but not detected in the graph, and as false positives if they were found in the graph but not in the ground truth.

### Simulated NGS dataset of *Neisseria meningitidis* for pangenome graph based variant calling

In addition to the comparative genomics analysis of the paths (genomes) based on the genome graphs, these graphs can also serve as a pangenome reference for NGS data analysis. To evaluate the genome graph-based pipeline for NGS data mapping and variant calling using the VG toolkit (Garrison et al., 2018), we simulated 100 × read depth 2 × 150 bp paired NGS data with an error rate of 0.5% using wgsim from samtools (Li et al., 2009).

We began with eight genomes, which included the 3ST genomes and the three simulated genomes, and two mutated genomes, ST41Mut and ST154Mut, based on the SNP difference of ST41 and ST154 relative to ST42. To generate a set of 40 genomes, we initially introduced 2000 SNPs for each of the eight genomes with five repeats, followed by two additional rounds of 2000 SNPs (40 genomes per round) using SimuG (Yue and Liti, 2019). Consequently, we obtained 128 genomes distributed among eight groups, including ST42, ST42Sim, ST41, ST41Mut, ST41Sim, ST154, ST154Mut, and ST154Sim.

### Real NGS dataset of NZMenB for pangenome graph based variant calling

To test the graph-based analysis for a real NGS dataset, we mapped the NGS dataset of 149 NZMenB isolates to the 3ST pangenome graph (Supplementary Table S2). The 149 isolates included 49 from clade154, 48 from clade41 and 52 from clade42.

### Map the NGS data to graph using the VG toolkit

To map the NGS data to genome graph using the VG toolkit, we initially converted graphs (4Sim and 3ST) into 256 bp chunks using the command “vg mod -X 256”. We then employed “vg index” to generate the index for the graph. Subsequently, “vg map” was utilized to map the NGS data against the graph, resulting in the generation of gam files. We also used ‘vg stats’ to check the mapping statistics.

To compare the mapping rates for NGS dataset against linear references *versus* the graph, we also mapped the NGS data to each linear reference using Bowtie2 version 2.3.2 (Langmead and Salzberg, 2012). All sequenced and aligned reads were further processed using the Picard-tools version 2.10.10 (https://broadinstitute.github.io/picard/) to remove duplicated reads and were assessed with Qualimap version 2.2.1 (Garcia-Alcalde et al., 2012).

### Variant calling for NGS data against genome graph

There are currently two popular approaches to call variants in pangenome graphs: genotyping known variants and novel variant calling. We utilized both methods to call variants for the 128 simulated NGS dataset against the 4Sim genome graph.

To genotype known variants in the graph, we employed “vg pack” to calculate the support reads for each gam file. We then utilized “vg call” to genotype the known variants for each sample based on the snarls file generated from the 4Sim genome graph.

To consider novel variants from the reads, we employed “vg augment” to augment each gam file. Subsequently, we indexed the augmented graph, calculated read support for all variants, and performed variant calling. High confidence variants were identified using the PASS information and genotype (GT = 1|1) from the VCF file. To evaluate the performance of variant calling in the context of simulated genomes, we compared the high confidence variants identified against the 4Sim graph with the simulated SNP records. As the actual variations of ST41 and ST154 relative to ST42 remain unknown, both sets of simulated NGS data were excluded from this analysis.

### Distance matrices for cluster relationship

To analyze the cluster relationship among the 130NM genomes, we utilized “odgi similarity” from odgi version 0.8.3 (Guarracino et al., 2022) to extract a sparse similarity matrix for paths of the 130 MN graph. We then converted the paired Jaccard similarities from column six into a Jaccard distance matrix. These Jaccard similarities are measures that represent the proportion of shared elements between pairs of paths. We then employed hierarchical clustering to construct the phylogenetic relationship among the genomes based on the Jaccard distances. To assess the accuracy of the clustering relationship, we compared it to the one obtained by kmer-based SNP phylogenetic analysis.

For the kmer-based SNP analysis, we used ska, a reference-free, contig-based analysis, to extract the SNPs derived from default kmer length 31 that were present in 90% of the isolates (Harris, 2018). Phylogenetic analyses were constructed from the kmer-based SNP alignment using maximum likelihood under the best-fit model by Bayesian Information Criterion with iqtree version 2.0.6 (Minh et al., 2020). The robustness of the clades was estimated with 2000 ultra-fast bootstrap replicates.

## Results

### Overview of the pangenome graph workflow

**FIGURE 1:**
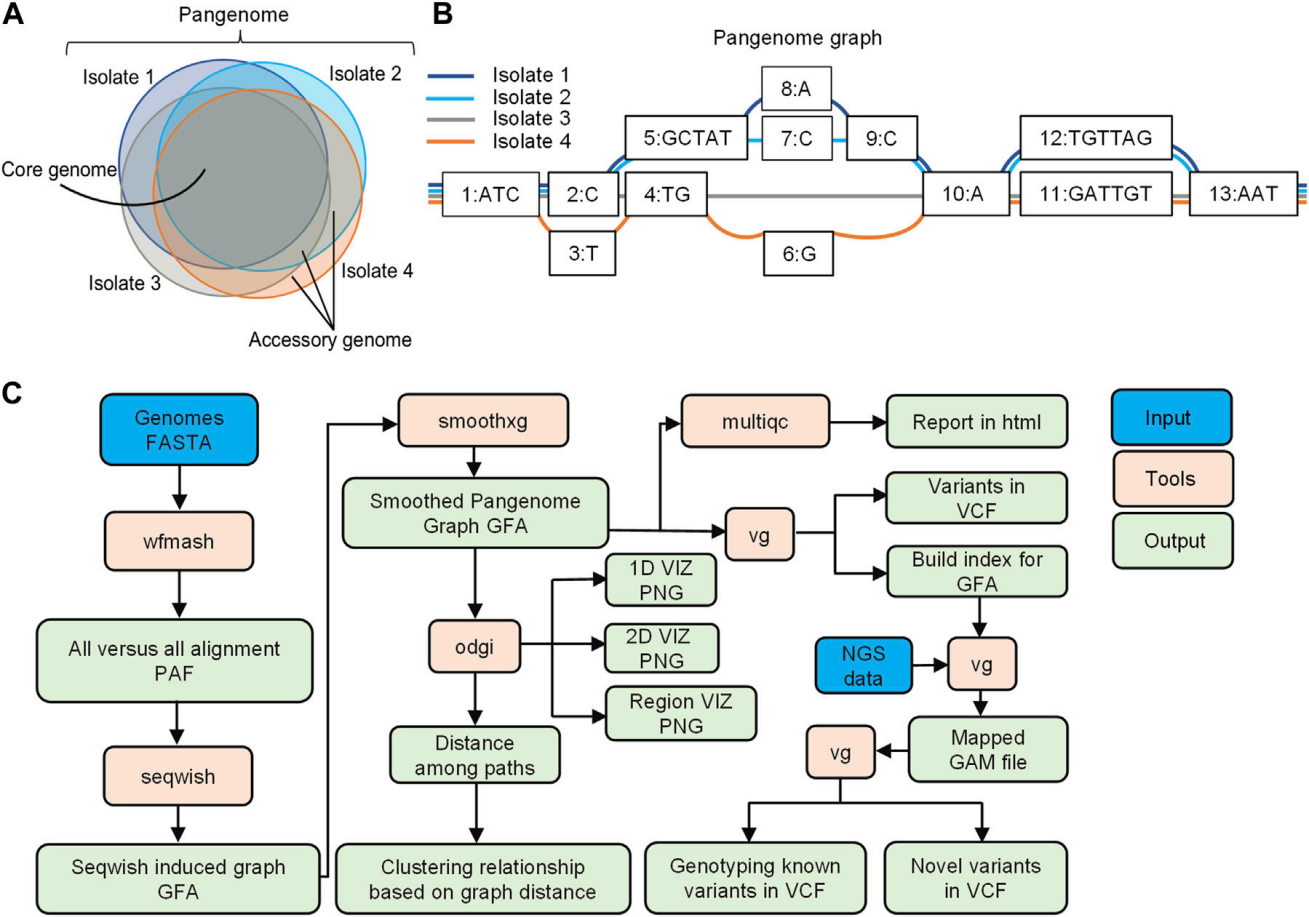
Overview of pangenome graph pipeline. **(A)** Bacterial pangenome, core genome, and accessory genomes. The pangenome represents the comprehensive collection of whole-genome sequences from multiple individuals within a clade. The core genomes comprise of a set of genes present in all individuals, while accessory genomes consist of genes found in a subset of individual genomes **(B)** Pangenome graphs representation. Pangenomes are stored in graph models, where nodes (numerically labeled) represent DNA segments of varying lengths. Edges connect nodes, and paths represent walks through the nodes of the graph, corresponding to the input genomes **(C)** Pangenome graph pipeline with PGGB. The pipeline includes graph construction using the PGGB tool, graph manipulation using ODGI, and variant calling for NGS data using the VG toolkit. The overview demonstrates an efficient and integrated approach to pangenome analysis.

A pangenome is defined as the comprehensive collection of whole-genome sequences from multiple individuals within a clade, a population or a species (Medini et al., 2005; Tettelin et al., 2005; Vernikos et al., 2015; Kavvas et al., 2018). This collective genomic dataset can be further divided into two distinct components: the core genome, which includes genes present in all individuals at the time of analysis, and the accessory genome, consisting of genes found only in a subset of individuals (Vernikos et al., 2015; Figure 1A). Pangenome graphs are pangenomes stored in graph models that can capture the entire genetic variation among genomes in a population or of a set of related organisms (Paten et al., 2017; Garrison et al., 2018; Eizenga et al., 2020; Garrison et al., 2023; Figure 1B).

In this study, we have developed a pangenome graph pipeline for microbial genomics, consisting of graph construction using PGGB (Garrison et al., 2023), graph manipulation through ODGI (Guarracino et al., 2022), and variant calling for NGS data using the VG toolkit (Garrison et al., 2018; Figure 1C). ODGI facilitates graph manipulation tasks such as visualization, and extraction of distances among paths in the graph, enabling phylogenetic analysis (Guarracino et al., 2022). Additionally, we utilized the VG toolkit for analyzing NGS data against the graph for read mapping and variant calling (Garrison et al., 2018).

The pangenome graph construction with PGGB was demonstrated to be effective across various datasets, though the resulting graphs varied significantly based on the complexity of the input genomes (Supplementary Table S3). The total run times for PGGB were 10.8 min, 8.3 min, and 4392 min, and the maximum memory usage was 1.87 GB, 2.01 GB, and 38.64 GB for the 4Sim, 3ST, and 130NM, respectively. In the case of the 130NM genomes, employing the PGGB tool with the “-x auto” option enabled for the giant component heuristic resulted in a total execution time of 2787 min and a peak memory usage of 21.92 GB. Notably, the generated graph remained identical to the one obtained without this option. In scenarios involving hundreds to thousands of genomes, it is recommended to utilize mapping sparsification (-x auto) to alleviate computational demands.

### High consistency between variations in the 4Sim genome graph and ground truth

**FIGURE 2:**
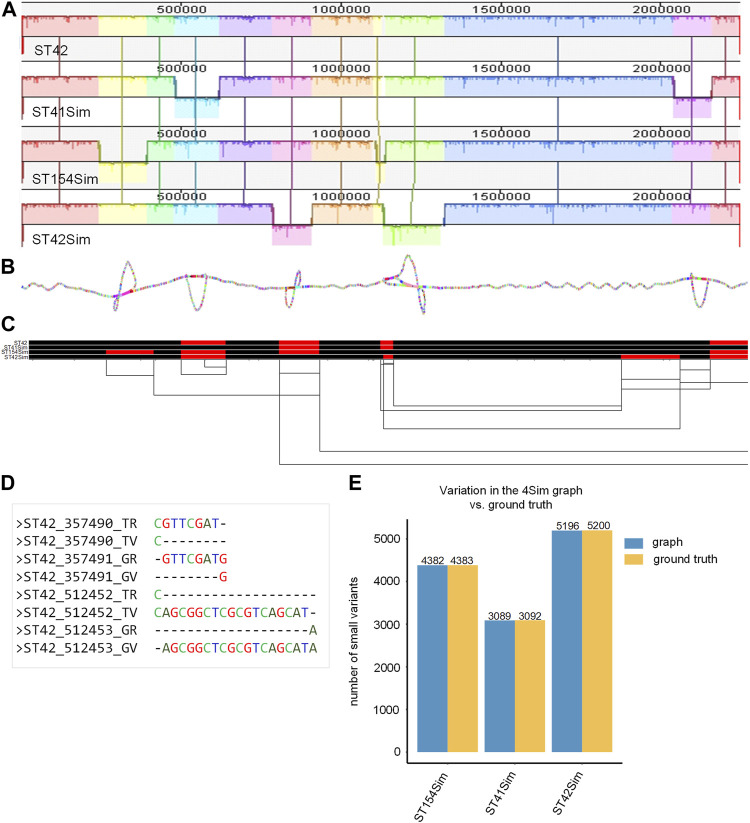
Pangenome graph of the 4Sim genomes. **(A)** Mauve alignment of the 4Sim Genomes. The Mauve alignment of the 4Sim genomes is depicted, with blocks under each line representing inverted regions **(B)** 2D visualization of the 4Sim pangenome graph. The pangenome graph of the 4Sim genomes is visualized in 2D using gfaestus. Bubbles in the graph indicate inversions. **(C)** 1D visualization of the 4Sim pangenome graph with path orientation, highlighting the inversions. The 4Sim pangenome graph is visualized in 1D using ODGI. Forward paths are represented in black, while reverse paths are in red. Regions displaying both black and red represent inversions **(D)** Inconsistency in indel region alignment: graph vs. ground truth. This panel provides two examples of inconsistent indel region alignment between the graph and the ground truth. For example, the deletion that appears at position 3547490 in ST42 according to the ground truth, is marked as being at position 3547491 in the graph. The labels are as follows: TR, true reference; TV, true variant; GR, reference in the graph; GV, variation in the graph. **(E)** Consistency of variations: graph vs. ground truth. A bar plot demonstrates the high consistency of variations between the graph and the ground truth, highlighting the accuracy of the pangenome graph representation.

The final smoothed graph for the 4Sim genomes spanned 2,260,981 bp and consisted of 30,033 nodes and 40,273 edges. This is slightly larger than each of the input genomes: 2,248,966 bp for NC_017518 (ST42); 2,249,014 bp for ST41Sim, 2,248,965 bp for ST154Sim, and 2, 249,050 bp for ST42Sim. Mauve alignment (Figure 2A) supported our observations, as inversions were displayed as bubbles in the 2D visualization (Figure 2B) and as inverted sequences in the 1D visualization (Figure 2C). The VCF file indicated that inversions were identified as different genotypes across various genomes. It is important to note that some variations in the graph did not correspond to the ground truth due to alignment discrepancies in the indel regions (Figure 2D). Upon manual inspection of these sites, it was found that these variants represented the same variation but were aligned to either the left or the right of the indels in the graph compared to the ground truth. We detected four, three, and two false negative small variations for ST154Sim, ST41Sim, and ST42Sim, respectively, in comparison to ST42. Additionally, we identified seven false positive small variants in ST154Sim. Therefore, both sensitivity and specificity for variations in graph compared to ground truth are over 99.9%. Despite the relatively simple nature of the simulated genomes, the agreement between the variations in the graph and the ground truth implies that the pangenome graph generated by PGGB is able to accurately and reliably detect genetic variant across the input genomes (Supplementary Table S4).

### 100% mapping rates and comparable variant calling in graph-based analysis of simulated NGS data

**FIGURE 3:**
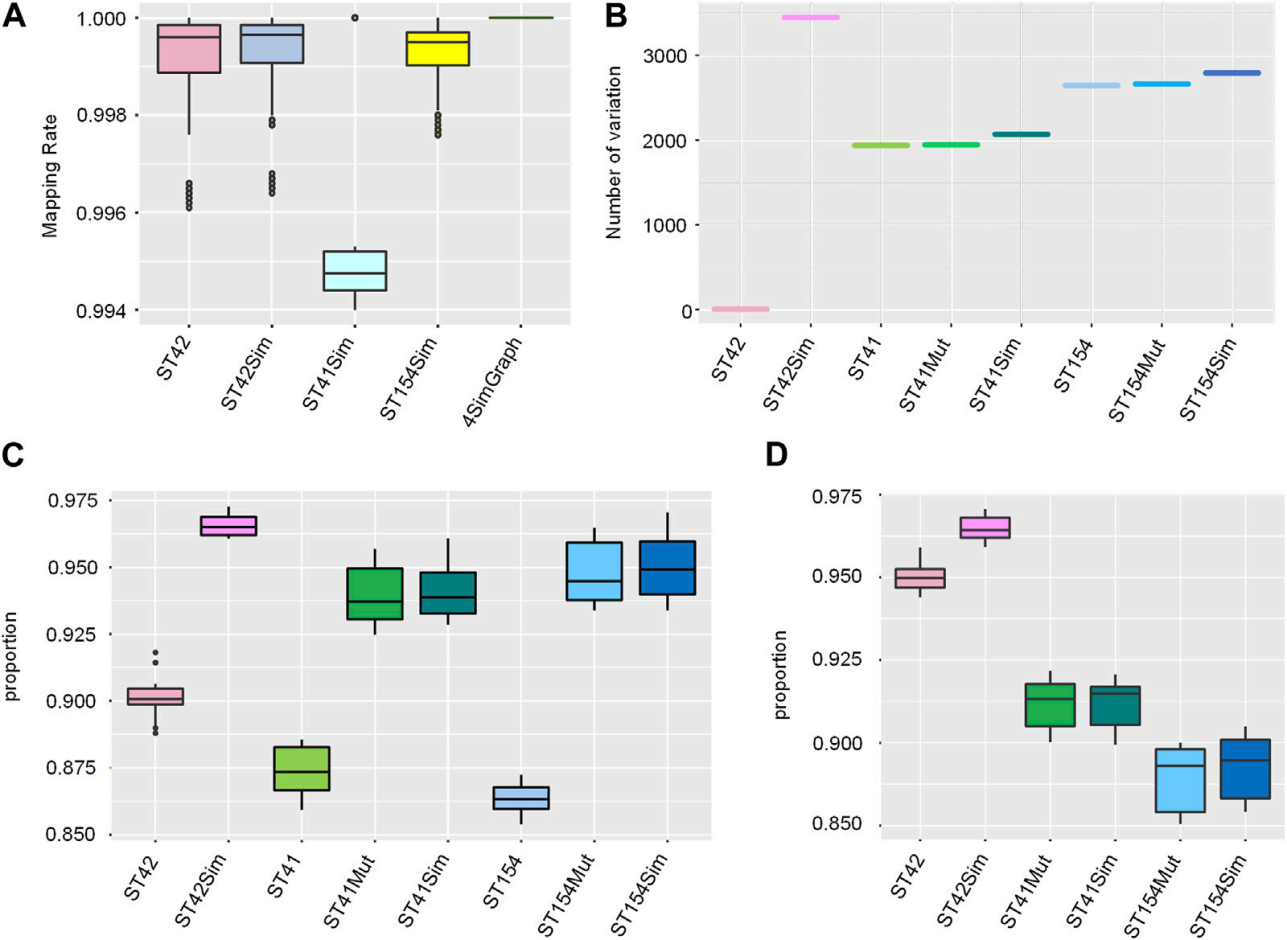
Mapping rates and comparable variant calling in graph-based analysis of simulated NGS Data. **(A)** Mapping rates: simulated NGS data to linear reference vs. 4Sim pangenome graph. This panel presents a comparison of mapping rates for simulated NGS data to each linear reference and the 4Sim pangenome graph. **(B)** Known variant genotyping in the 4Sim graph. A box plot displays the number of variations in genotyping based on known variants within the 4Sim graph. **(C)** Novel variants from graph-based calling. The box plot shows the proportion of high-confidence variants for each simulated group of data, illustrating the effectiveness of graph-based variant calling **(D)** Overlap of high-confidence variants with simulated variants. This box plot represents the proportion of high-confidence variants that overlap with simulated variants for each group, demonstrating the accuracy of graph-based variant calling in identifying true variations.

Utilizing a pangenome graph reference for the analysis of NGS data can significantly enhance mapping rates. We conducted an evaluation by comparing the mapping rates of simulated NGS data based on the 4Sim graph to each of the linear references: ST42, ST42Sim, ST41Sim, and ST154Sim. All datasets were mapped to the graph, yielding a 100% mapping rate. Although the rates of NGS data aligned to each single linear reference were all over 99%, a bias was observed in the linear reference mapping rates (Figure 3A). Our findings indicate that the use of a pangenome graph reference can greatly improve mapping rates in NGS data analysis.

The pangenome graph integrates various genomic variants, making it possible to genotype variants in NGS datasets. Interestingly, the genotyped results demonstrated high consistency across the eight simulated NGS datasets (Figure 3B; Supplementary Table S6). The ST42Sim group exhibited the highest number of variants, consistent with the original simulation of 5000 SNPs and 200 indels. Moreover, the ST41Sim group displayed more identified variants compared to ST41 and ST42Mut, while the ST154Sim group revealed more variants compared to ST154 and ST154Mut.

Variant calling for NGS data against the graph using the VG toolkit differs slightly from single linear reference-based calling. In the absence of a defined path for variant calling, the process will call variants against the paths in alphabetical order (e.g., core genome part from A path, accessory genomes from B path, etc.). The variant call format file includes a PASS column to mark variants that pass all filters, and the genotype (GT) describes the identified genotype in each sample. Since we analysed haplotype bacterial genomes, variants with PASS but GT not equal to 1|1 were classified as errors, while those with PASS and GT = 1|1 were classified as high-confidence variants. For each simulated NGS group, high-confidence variants exhibited consistency. Interestingly, the ST41 and ST154 groups exhibited the lowest proportion of high-confidence variant calls, which may be attributed to these groups’ greater genomic diversity and the absence of a reference from either group in the graph. Including one reference from each of these groups in the pangenome graph led to an improvement in the proportion of high-confidence variant calls (Figure 3C; Supplementary Table S8). Furthermore, as NC_017518 (ST42) was the first path from the graph for variant calling, the ratio of high-confidence variants to the number of simulated variants was higher in ST42 (0.944–0.959) and ST42Sim (0.959–0.9706), but relatively lower in ST154Mut (0.8755–0.9000) and ST154Sim (0.8792–0.9049) (Figure 3D).

### Enhanced mapping of NZMenB real NGS data to pangenome graph

**FIGURE 4:**
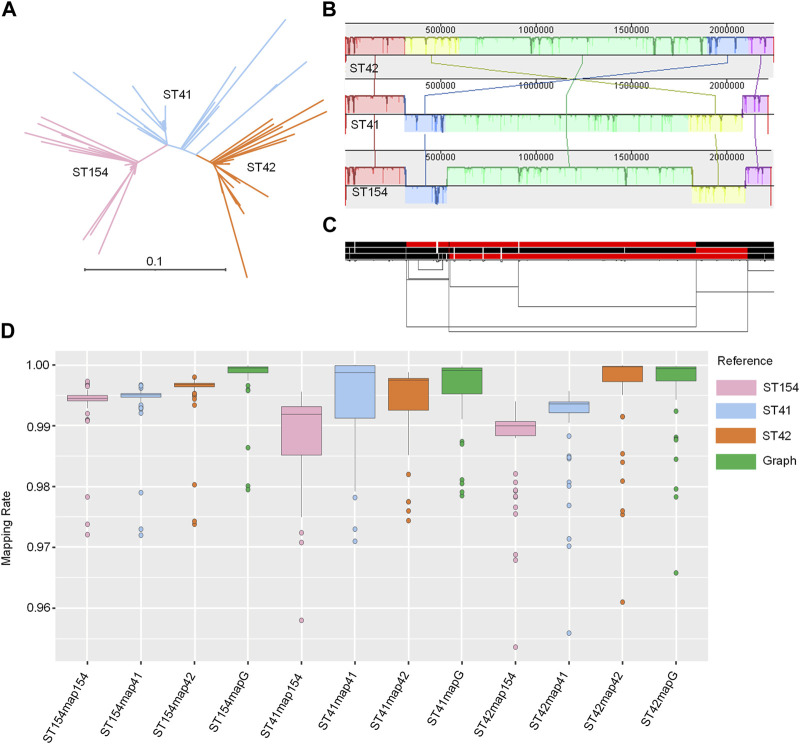
Pangenome graph of 3STs *N. meningitidis* genomes. **(A)** Phylogenetic analysis of NZMenB. The phylogenetic analysis of NZMenB reveals three major STs responsible for the epidemics: ST154, ST41, and ST42 **(B)** Mauve alignment of 3ST genomes. The Mauve alignment of the 3ST genomes is depicted, with blocks under each line representing inverted regions. **(C)** 1D visualization of the 3STs pangenome graph with path orientation. The 3STs pangenome graph is visualized in 1D using ODGI, displaying path orientation. Forward paths are represented in black, while reverse paths are in red. Regions displaying both black and red represent inversions **(D)** Mapping rates: real NZMenB NGS data to linear reference vs. 3STs to the pangenome graph. This panel presents a comparison of mapping rates for real NZMenB NGS data to each linear reference and the 3STs pangenome graph. Each group, ST154, ST41, and ST42, were mapped to their respective references and the graph.

The three sequence types (STs) represent the three major clades of NZMenB (Figure 4A). The final graph for 3STs spanned 2,304,073 bp, consisting of 23,323 nodes and 31,325 edges. This is marginally larger than each of the input genomes: 2,248,966 bp for NC_017518 (ST42); 2,217,832 bp for NMI01191 (ST41) and 2,233,582 bp for NMI97348 (ST154). The inverted regions are consistent in both the Mauve alignment (Figure 4B) and the 1D graph visualization (Figure 2C).

We mapped each group of genomes (ST154, ST41, and ST42) to the respective linear references - ST154, ST41, ST42, and the 3STs graph. Despite the higher diversity of the compared genomes, particularly within the ST41 group, the mapping rate was higher when mapped to the graph as opposed to a single linear reference (Figure 4D; Supplementary Table S9). For example, when examining the reads of ST154 and their mapping to the ST154, ST41, ST42, and 3ST genome graphs, we observed values ranging from 0.9721 to 0.9973, 0.972 to 0.9967, 0.9738 to 0.998, and 0.9795 to 0.9999, respectively. The isolates of the ST154 group may be less diverse, as indicated by the smaller range of mapping rate variation, while the isolates of the ST41 group display greater diversity, as evidenced by the larger ranges of mapping rate variation (0.958–0.9956, 0.971 to 1, 0.9744 to 0.9988, and 0.9785 to 0.9998, respectively). The isolates belonging to the ST42 group displayed comparable mapping rates when mapped to both the ST42 and 3ST genome graphs. However, slightly lower mapping rates were observed when these isolates were mapped to ST154 (ranging from 0.9536 to 0.994) and ST41 (ranging from 0.9559 to 0.9959). In summary, these findings suggest potential reference bias when using a single linear reference and demonstrate that utilizing a graph as a reference can improve the mapping process.

### The clustering relationships among paths in the genome graph effectively reveal phylogenetic connections

We evaluated the performance of the PGGB method on a diverse group of 130NM genomes, constructing a pangenome graph that proved more complex than the 4Sim and 3ST pangenomes. The 130NM pangenome graph spans 4,751,450 base pairs, over twice the size of a typical individual *N. meningitidis* genome and comprises 629,349 nodes and 894,725 edges.

**FIGURE 5:**
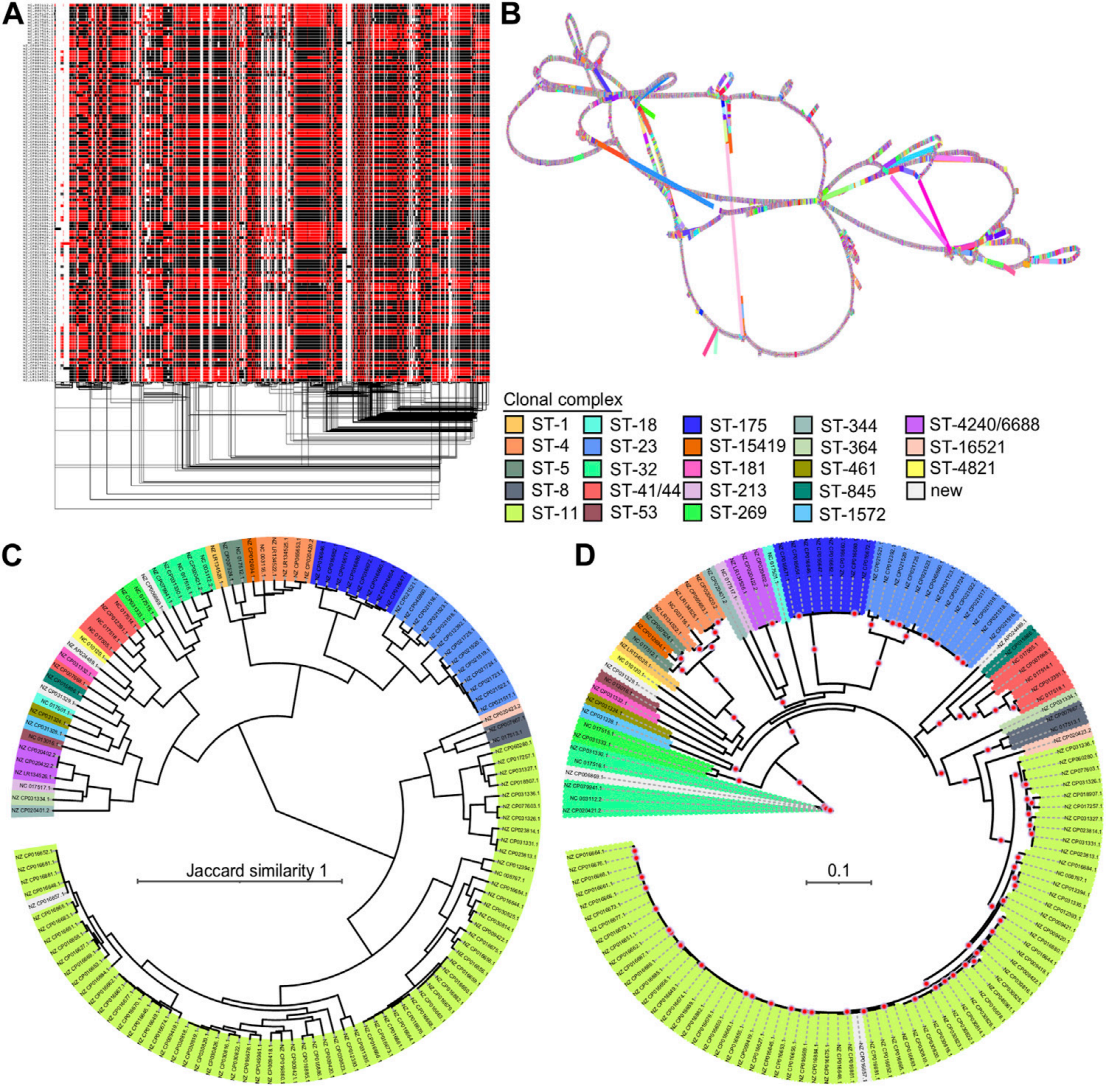
Pangenome graph of 130 *N. meningitidis* genomes and their phylogenetics relationships. **(A)** 1D visualization of the 130NM genomes with path orientation. The 130NM pangenome graph is visualized in 1D using ODGI, displaying path orientation. Forward paths are represented in black, while reverse paths are in red. Regions displaying both black and red represent inversions **(B)** 2D visualization of 130NM pangenome graph. The pangenome graph of the 130NM genomes is visualized in 2D using gfaestus. **(C)** Phylogenetic analysis of 130 NM genomes based on Jaccard distance of paths. The clustering relationship of 130 NM genomes is conducted based on Jaccard distance of paths in the 130NM pangenome graph. Isolate names and clades are coloured according to their clonal complex designation, with “New” indicating isolates where the clonal complex is not yet assigned **(D)** Phylogenetic analysis of 130NM genomes based on kmer SNPs. A maximum-likelihood phylogeny is constructed using iqtree v.2.0.6 under the best-fit model determined by the Bayesian Information Criterion. Branches with greater than 95% bootstrap consensus (from 2000 ultra-fast bootstrap replicates) are highlighted with a red dot. Isolate names and clades are coloured according to their clonal complex designation.

The 1D visualization of the 130NM graph, which colours paths based on orientation, shows genome chunks as either forward (black) or reverse (red) (Figure 5A), illustrating the high recombination rate of *N. meningitidis* genomes. The 2D visualization using gafestus reveals large bubbles, potentially due to the substantial number of genomes aligned in reverse (Figure 5B). We classified the variations in the graph into (multiple) SNPs, indels and SVs. An example of a multiple nucleotide polymorphism (MNP) is when a sequence variation involves changes in multiple adjacent nucleotides. For example, a DNA sequence changes from “GGG” to “ACA”. The 130NM pangenome graph contains 133, 745 (M) SNPs, 25,478 indels, and 1,446 SVs.

The all-vs-all alignment pangenome graph construction is unbiased, allowing distances among paths in the graph to effectively reveal genome relationships. Using the Jaccard similarity of the 130NM paths, we constructed a phylogenetic relationship among them. Clonal complexes are well-resolved by Jaccard similarity, with groups containing more than one genome clustering together (Figure 5C). This finding is largely consistent with phylogenetic relationships revealed by the kmer SNP-based analysis (Figure 5D). Most of the highly supported clades identified by the kmer SNP-based analysis are also clustered together on the Jaccard similarity tree, such as clonal complex ST8, ST23, ST175, ST420/6688, ST4, ST269, ST41/44, but the branches in the kmer SNP-based analysis are more diverse. There are two clonal complexes, ST-344 and ST-32, being clustered together on the Jaccard similarity tree but not on the kmer SNPs tree. Overall, the all-vs-all alignment pangenome graph is suitable for a relatively large number of genomes, capturing all types of variation and offering an unbiased method for genome comparison. The distance of pangenome graph paths reveals the genomic relationships well.

## Discussion

Whole genome sequencing has revolutionized many aspects of infectious disease research, including the tracking and monitoring of pathogen spread and evolution (Didelot et al., 2012; Quick et al., 2016; Gardy and Loman, 2018; Geoghegan et al., 2020; Geoghegan et al., 2021; Yang et al., 2021), identification of drug susceptibility and resistance (Koser et al., 2014; Holt et al., 2015; Walker et al., 2015), and the advancement of vaccine development (Chen et al., 2021). However, the use of linear reference-based approaches for genomic analyses may lead to biases, particularly in studies focused on highly variable bacterial genomes (Darmon and Leach, 2014). To overcome the limitation of single linear reference genomes, pangenome graphs offer an efficient model for representing and analyzing multiple genomes and their variants within a graph structure encompassing all types of variations (Paten et al., 2017; Eizenga et al., 2020). In this study, we present a practical and unbiased bioinformatic pangenome graph pipeline (Figure 1C) that uses PGGB to construct pangenome graphs from assembled genomes for comparative genomics (Garrison et al., 2023), and employs the VG toolkit to align whole genome sequencing data and call variants against a graph reference (Garrison et al., 2018). We have demonstrated the efficacy of the pangenome pipeline across a diverse collection of *N. meningitidis* genomes, using both simulated and actual genomic datasets.

Numerous methods exist for constructing pangenomes, each with specific strengths and strategies (Armstrong et al., 2020; Gautreau et al., 2020; Li et al., 2020; Colquhoun et al., 2021; Ekim et al., 2021; Hickey et al., 2023); however, the PanGenome Graph Builder (PGGB) distinguishes itself by providing a comprehensive, unbiased approach that includes all types of genomic variations and treats each input genome equally (Guarracino et al., 2023). Using PGGB, we have successfully constructed pangenome graph for diverse datasets of *Neisseria meningitis* (the 4Sim, the 3STs and 130NM datasets). The resulting graphs varied considerably based on input genome complexity (Supplementary Table S3). The resulting pangenome graph proved to be a powerful tool for visualizing and analyzing the complex genomic relationships among these highly recombinant *Neisseria* genomes (Figures 2, 4, 5). By capturing all types of genomic variation and enabling unbiased genome comparisons, this approach offers significant advantages for comparative genomics studies. The accurate representation of inversions, SNPs, and indels in the graph for the 4Sim genomes (Figure 2; Supplementary Table S4) serves as strong evidence for the effectiveness of PGGB. Moreover, the flexibility offered by PGGB to adjust parameters according to the user’s dataset is noteworthy. When using PGGB for pangenome graph construction, one can specifically adjust essential parameters such as -n, -s, and -p. These adjustments provide tuning opportunities to generate optimized graphs for different input datasets (Guarracino et al., 2023). In addition, enabling the -x auto option, the heuristic based on a model of random graphs to set a sparsification threshold for initial mappings of the 130NM dataset can significantly reduce computational time and maximum memory usage, but without compromising the accuracy of the final pangenome graph results (Supplementary Table S3).

In addition to representing various types of genomic variation in the pangenome graph generated by PGGB, we can also utilize distance metrics, such as the Jaccard distance of paths in the graph, to examine genomic relationships. Strains of *N. meningitidis* were classified into distinct clonal complexes based on similarity of STs by MLST (Maiden et al., 1998), reflecting their close evolutionary relationships. However, the high recombination rate of meningococcal genomes complicates the interpretation of phylogenetic relationships among strains and clonal complexes, and there is a need for novel genomic approaches to better understand their evolution (Harrison et al., 2017). For the diverse 130NM genomes, most highly supported clades identified by the kmer SNP-based analysis were also clustered together on the Jaccard distance tree (Figures 5C, D). This consistency underscores the utility of the pangenome graph approach for uncovering the underlying genomic relationships among *N. meningitidis* strains. Interestingly, we observed that the branches in the kmer SNP-based analysis are more diverse, suggesting that combining different methods of analysis may provide a more comprehensive understanding of the phylogenetic relationships among clonal complexes.

To circumvent reference bias, utilizing a pangenome as a reference is a significant direction for future genomics studies. In addition to pangenome graph construction using the PGGB method, our pipeline also employs the VG toolkit for the analysis of NGS data, which includes mapping and variant calling. Both simulated NGS and real data demonstrate improved mapping rates when using graph-based references compared to linear references, indicating that the adoption of a pangenome graph reference can substantially enhance mapping rates in NGS data analysis (Figure 3A; Figure 4D). The pangenome graph effectively integrates various genomic variants, enabling the genotyping of variants in NGS datasets that cannot be achieved using a single linear reference (Figure 3B). Furthermore, the novel variant calling approach based on the graph provides increased flexibility, allowing for either pangenome-based or reference-based variant calling. This feature significantly reduces reference bias and improves data analysis efficiency. Our results also reveal that the proportion of novel variant calls is remarkably high (Figure 3C), and a large number of simulated variations are identified (Figure 3D), indicating the reliability of graph-based NGS data analysis.

The incorporation of unbiased pangenome graphs into infectious disease research represents a remarkable advancement, yielding numerous benefits. Our pipeline employs PGGB for pangenome construction, which treats all input genomes in tandem, giving equal importance to every base. This comprehensive approach allows us to discern all genetic variation particularly structural variation and copy number variation that were likely overlooked by previous methodologies based on the use of a single reference genome. This enhanced detection capability proves crucial for the identification of virulence and antimicrobial resistance genes (Ekim et al., 2021). Simultaneously including all variations enhances our understanding of the genomes’ evolutionary history, helping elucidate transmission patterns and establish connections between cases. This could prove invaluable in infectious disease research, where identifying the source or potential origins of new outbreaks is a priority, rapid genotyping against a graph could offer essential clues. Moreover, with multiple genomes integrated into the graph, each genome or the entire pangenome can serve as a reference for novel variant calling. This feature becomes especially valuable in public health surveillance, eliminating the need to try different references. This unbiased pangenome graph approach holds the potential to address longstanding challenges in infectious disease research, such as the origin of antibiotic resistance, a concern with significant public health implications. Pangenome graphs can be used to track and understand the genetic determinants contributing to resistance, providing insights that could guide the selection of therapeutic modalities or preventive strategies. They allow researchers to visualize and interpret the complex genetic interactions and variations that propel the evolution of pathogenicity, thereby fostering a deeper understanding of pathogen behavior (Zhou et al., 2020). Additionally, they can elucidate processes such as horizontal gene transfer and evolution of genome architecture, both crucial for bacterial adaptability and survival (Soucy et al., 2015). Pangenome graphs are particularly beneficial for viral genomics studies, as viral genomes are smaller. The unbiased analysis of these genomes could provide evidence about their origin and spread, guiding the design of better vaccinations, and enhancing our ability to prevent, monitor, and treat infections.

Although the concept of pangenomes initially emerged from microbial research (Medini et al., 2005; Tettelin et al., 2005; Vernikos et al., 2015; Kavvas et al., 2018), pangenome graphs have since been applied to various species, such as humans (Guarracino et al., 2023; Liao et al., 2023), and cattle (Talenti et al., 2022). Integrating all genomic variants facilitates a comprehensive and unbiased view of the genetic landscape, as demonstrated by the draft human pangenome that not only captures known variants, haplotypes, and new alleles at complex loci but also adds 119 million base pairs of polymorphic sequences and 1,115 gene duplications compared to the existing GRCh38 reference (Liao et al., 2023). The research conducted by Guarracino et al. (2023) using PGGB confirmed a long-held hypothesis regarding the evolution of human acrocentric chromosomes—that these chromosomes contain pseudo-homologous regions where heterologous pairs recombine as if they were homologs. Pangenome graphs hold potential in the broader field of genomics, including human genetics and personalized medicine, where they can help uncover subtle genetic variations associated with disease susceptibility or treatment response. These methods are also expected to find applications in metagenomics, transcriptomics, and epigenomics, aiding in a more comprehensive understanding of genomic diversity.

In conclusion, the current pangenome pipeline has several advantages over other pipelines, offering a more comprehensive and accurate approach for comparative genomics and comprehensive genetic variation analysis for infectious disease. Pangenome graphs provide a promising and practical approach for advancing our understanding of pathogen diversity, evolution, and adaptation.

## Data Availability

The 4Sim, 3ST, 130NM datasets used for pangenome graph construction, 128 simulates genomes, and the scripts used in this study are available at https://github.com/ZoeYang2020/Pangenome-Graphs-in-Infectious-Disease. 149 isolates of NZmenB NGS data are available for download from the National Center for Biotechnology Information Sequence Read Archive (Bioproject accession no. PRJNA592848 and PRJEB28859). All the related materials are already publicly available.
